# Cellular Responses to Hydrophobic Polyelectrolyte/Wax Coatings for Biomedical Use

**DOI:** 10.1021/acsomega.5c06604

**Published:** 2025-11-24

**Authors:** Tonya D. Andreeva, Kiriaki Athanasopulu, Anita Lorenz, Ole Jung, Mike Barbeck, Rumen Krastev

**Affiliations:** † Faculty Life Sciences, 64332 Reutlingen University, Alteburgstraße 150, 72762 Reutlingen, Germany; ‡ Clinic and Policlinic for Dermatology and Venereology, University Medical Center Rostock, Strempelstraße 13, 18057 Rostock, Germany; § Natural and Medical Sciences Institute at the University of Tübingen (NMI), Markwiesenstraße 55, 72770 Reutlingen, Germany; ▼ Institute of Biophysics and Biomedical Engineering, Bulgarian Academy of Sciences, Acad., G. Bonchev Str., Bl. 21, 1113 Sofia, Bulgaria

## Abstract

Polyelectrolyte multilayer (PEM) coatings represent a promising strategy for the biofunctionalization of biomaterials. Incorporating nonpolymeric components into the polymer matrix is a strategy to modulate PEM properties, enabling the development of new, application-specific functionalities. For example, integrating nano-thick wax layers both within and atop the PEM matrix creates hydrophobic waterproof barrier coatings that show great potential for use on bioresorbable magnesium implants. These coatings hinder contact between the implant and surrounding tissue and bodily fluids, thereby slowing down implants’ degradation. However, the hydrophobic nature of such coatings raises concerns regarding their cell compatibility and overall biocompatibility. This study investigates and compares the viability of fibroblasts (3T3 cells) and human umbilical vein endothelial cells (HUVECs) on three different hydrophilic PEM coatings and their corresponding composite hydrophobic PEM/Wax counterparts. Our results show that some aspects of cellular behavior are cell-type-specific, while others are commonly influenced by the chemical composition of the coatings. Although hydrophobic, all three PEM/Wax coatings supported fibroblast adhesion and growth, surpassing their hydrophilic PEM counterparts, whereas the more surface- and environment-sensitive HUVECs showed reduced adhesion and viability on the hydrophobic coatings compared with the hydrophilic PEMs. Cytotoxicity testing, conducted in accordance with ISO 10993-5, confirmed that all coatings are noncytotoxic, supporting their suitability for use in medical devices.

## Graphical abstract

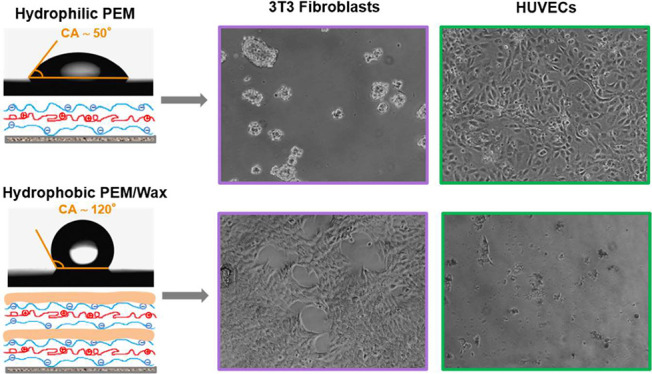

## Introduction

Year after year, the global market for medical devices continues to experience remarkable growth.^1^ One of the major factors driving this expansion is the increasing prevalence of an aging population worldwide, which contributes to the rising demand for medical equipment. As the variety of products on the market grows, so too does the need to develop new coatings for these devices, particularly since up to 80% of medical devices require at least one type of coating or surface treatment. This demand is also reflected in the significant growth in the market for medical device coatings.

Functional medical coatings are a class of materials that, when applied to the surface of a medical device, provide additional functionality or improve its biocompatibility. These coatings act at various interfaces, such as the coating-surrounding environment, the substrate-coating interface, or even within the bulk of the coating. Typical members of this specialized coatings, which add significant value to the functionality of medical devices and are ranked according to their market, include: hydrophilic coatings, antimicrobial coatings, hydrophobic coatings, antifriction coatings, bioresorbable coatings, osseointegrating coatings, lubricating coatings, drug-delivery coatings, and anticorrosion coatings.

Hydrophobic coatings hold the third position in the global medical device coatings market.^2^ They are used across a wide range of medical devices, including surgical instruments and implants. Their importance is growing due to their key role in infection control as well as in enhancing device performance and durability. When applied, hydrophobic coatings provide fluid-repellent, self-cleaning, antifouling, and anticorrosive properties, all of which help to reduce the risk of contamination and infections in patients.

Hydrophobic polytetrafluoroethylene (PTFE) coatings have long been integral to medical devices due to their exceptional chemical resistance, low friction, and biocompatibility.^3^ However, the medical industry is currently experiencing a significant shift in PTFE coating technologies, driven by increasing regulatory scrutiny over perfluoroalkyl and polyfluoroalkyl substances (PFAS). Due to environmental and health concerns, agencies like the U.S. Environmental Protection Agency (EPA) and the European Union have imposed restrictions on these substances, prompting manufacturers to seek alternative solutions. Wax-based coatings represent a promising alternative and are widely employed across various industries – including automotive (antirust materials),^4^ food (fruit, vegetable, and cheese coatings),^5,6^ textile (waterproofing textiles),^7^ and paper (paper packaging) sectors^7,8^ – owing to their hydrophobicity, barrier-forming capacity, and biocompatibility. Wax-coated polymer systems show considerable promise for biomedical applications, particularly for their ability to enhance surface hydrophobicity, regulate degradation kinetics, and improve barrier performance.^9,10^ Wax-based coatings have been successfully applied in pharmaceutical contexts, where natural waxes and alginate-fatty glyceride composites provide superior gastro-resistance for oral drug delivery.^11,12^ In waterborne polymer coatings, the incorporation of biobased waxes has also been shown to enhance moisture resistance and controlled-release behavior, further underscoring their functional versatility.^13^ Moreover, broader reviews of biodegradable polymers emphasize the importance of surface modifications, such as wax-based barriers, for tailoring biocompatibility and degradation kinetics of implantable devices.^14^ Collectively, these studies highlight the adaptability of wax-coated polymer systems across a range of biomedical applications, justifying further exploration of their use in cell-interfacing materials and implantable platforms.

The concept that the adsorption of charged paraffin particles onto polyelectrolyte multilayers (PEM) can inhibit water diffusion was first proposed by Glinel et al.^15^ Building on this idea, our recent publication described the construction of composite PSS/PAH/Wax films (PSS - polystyrenesulfonate, PAH -poly­(allylamine hydrochloride)) incorporating three different types of wax, and demonstrated their anticorrosion properties. These films showed great promise as protective coatings for magnesium (Mg) alloy-based bioresorbable implants.^9^ Specifically, we reported that applying a PSS/PAH/Wax coating to the surface of a marketed Mg-based cardiovascular stent significantly reduced the degradation rate compared to uncoated Mg-based stents. The coatings were constructed using the layer-by-layer (LbL) deposition technique, which involves the sequential deposition of oppositely charged polyelectrolytes (PSS and PAH) from aqueous solutions to form the base PEM. Wax nanoparticles, suspended in water or ethanol, were then adsorbed onto the multilayer structure. This composite was subsequently subjected to an annealing process, during which the wax particles melted and formed a continuous nanometer-thick wax layer.

Composite PEM/Wax coatings offer several distinct advantages over conventional hydrophobic waterproof coatings, particularly in biomedical and implant-related applications. PEM films provide a highly tunable platform, allowing precise control over surface chemistry, charge, thickness, and mechanical stiffness.^16^ By carefully selecting and layering specific polyelectrolytes, these coatings can be engineered to modulate key biological interactions such as protein adsorption, cell adhesion, and drug release.^16^ The LbL self-assembly technique used to fabricate PEMs enables nanometer-scale precision, far exceeding what can be achieved with bulk coatings or spray-on hydrophobic films. Moreover, this method allows for the integration of wax layers not only on top of but also within the PEM structure, resulting in multifunctional surfaces with tunable properties.^9,11^ This modularity gives PEM/Wax composites a clear advantage over traditional, single-function hydrophobic layers. We recently reported, for the first time, that PEM coatings exhibit strong adhesion to titanium substrates (analyzed according to ASTM F-1147-5 standards) and meet the ISO requirements for coatings on metal implants.^17^ The PEM matrix acts as a cushion that improves the adhesion of wax particles, resulting in enhanced coating stability, especially under physiological conditions. In contrast, single wax coatings frequently suffer from poor adhesion, cracking, or delamination when exposed to the complex biochemical environment of the body. These limitations can compromise the protective barrier, leading to the premature degradation of the underlying implant. Furthermore, single wax layers may fail to provide uniform coverage over complex geometries, leaving areas vulnerable to fluid infiltration and corrosion.

The hydrophobicity of these coatings, however, raises questions regarding their biocompatibility and cell compatibility. Generally, hydrophobic surfaces are considered to inhibit cell attachment and growth. Most mammalian cells prefer moderately hydrophilic surfaces with contact angles ranging from 40 to 70° for adhesion and growth.^18^ For instance, fibroblast adhesion and proliferation are more strongly stimulated by hydrophilic surfaces like clean glass and aminopropylsilane than by hydrophobic surfaces like silicone and polylactate.^19^ Patterning of a hydrophobic cyclic olefin copolymer substrate (with a contact angle of 110°) with oxygen plasma treatment and graphene oxide (both hydrophilic) results in MDA-MB-231 cancer cell adhesion and proliferation being restricted to the hydrophilized areas only.^20^ Another study demonstrated a novel cell patterning approach, showing that epithelial cells do not adhere to a hydrophobic PDMS surface, but adhere easily to a plasma-treated hydrophilic PDMS surface.^21^

This work investigates the ability of three different hydrophobic composite PEM/Wax coatings to support the attachment and growth of 3T3 fibroblasts and human umbilical vein endothelial cells (HUVECs). The coatings consisted of alternating polymer matrices and nano-thick wax layers. The polymer matrices were composed of various PEMs, built through the self-assembly of weak and strong polyelectrolytes. The base PEMs possess inherent surface properties that can also influence the cellular behavior. Therefore, we explored the potential to regulate fibroblast and endothelial cell adhesion and growth by combining different PEMs with wax layers.

## Materials and Methods

### Materials

Polyelectrolytes–poly­(ethylene imine) (PEI, 750 kDa, 50 wt %); PSS (70 kDa); poly­(acrylic acid), PAA (100 kDa, 35 wt %); and chitosan, Chi (50–190 kDa, 75–85% deacetylated) were all purchased from Sigma-Aldrich (Steinheim, Germany). PAH (120–200 kDa) was obtained from Alfa Aesar (Thermo Fisher (Kandel) GmbH), and hyaluronic acid (HA, 360 kDa), from Lifecore Biomedical, LLC (Chaska). All of the polyelectrolytes were used as received. PSS, PAH, and PAA were dissolved in 0.5 M NaCl to a concentration of 2 mg/mL and adjusted to pH 7.0. HA and Chi were also dissolved in 0.5 M NaCl, at a concentration of 1 mg/mL, and adjusted to pH 5.5. PEI was dissolved in ultrapure water to a concentration of 2 mg/mL and adjusted to pH 7.0. An aqueous wax suspension (25% w/w), containing anionic, paraffin-based spherical particles, was purchased from Keim-Additec Surface GmbH (Germany) and resuspended to a final concentration of 3% w/w. The wax particles had a melting range of 56–85 °C (as listed by the supplier), a diameter of 65.2 ± 0.5 nm, and a zeta potential of −44.8 ± 2.9 mV (as measured with dynamic light scattering).

For physicochemical characterization, the coatings were constructed on silicon (100) wafers (10 × 10 mm^2^, CrysTec GmbH, Germany) precleaned by successive ultrasonication in acetone and isopropanol (2 min each). For cell culture experiments, identical coatings were prepared inside sterile 24-well cell culture plates (Corning Inc., New York, USA).

### Building of the PEM and Composite PEM/Wax Coatings

The coatings were constructed by the LbL deposition technique involving polyanions, polycations, and charged wax particles, as schematically shown in Figure 1, adopting the technology described in refs 1,15 . Briefly, the substrate was first primed with a single layer of PEI, followed by the construction of the base PEM (Figure 1a). A monolayer of charged wax nanoparticles was then deposited by adhesion from a wax suspension. This hybrid structure was subsequently washed with water and annealed in a laboratory oven (Heratherm, Thermo Scientific) for 45 min at 90 °C. As a result, a nanothin, homogeneous wax layer formed on top of the PEM (Figure 1c). The resulting PEM film covered with a thin wax layer represents one building block of the composite PEM/Wax coating. The entire cycle from (a) to (c) was repeated to construct the final (PEM/Wax)_2_ coating, consisting of two building blocks (Figure 1d). Three types of PEMs composed of different polyelectrolyte pairs were applied as base coatings: (HA/Chi)_7_, (PAA/PAH)_7_, and (PSS/PAH)_7_. The number of seven bilayers was selected to ensure the formation of continuous and homogeneous PEM films, as a minimum of six bilayers is required for film integrity.^22^ All PEM coatings were annealed under the same conditions as those of their corresponding composite PEM/Wax counterparts. The composite PEM/Wax multilayers investigated in this study are termed HA/Chi/Wax, PAA/PAH/Wax, and PSS/PAH/Wax. Each type of coating was prepared and tested in at least three independent replicates.

**Figure 1:**
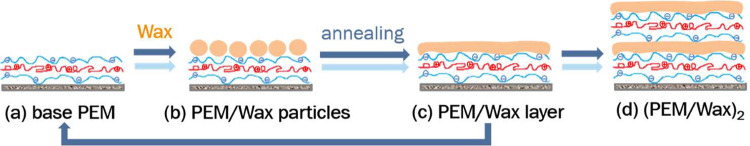
Schematic representation of the construction process of composite (PEM/Wax)_2_ multilayers.

For cell culture experiments, all coatings were prepared under sterile conditions using sterile filtered solutions (bottle-top filters with SFCA membrane, pore size of 0.45 μm, Carl Roth GmbH, Germany).

### Physicochemical Characterization of the Base PEM and Composite PEM/Wax Coatings

The thickness of the coatings was measured by spectroscopic ellipsometry (Sentech, Germany) in the dry state. Hydrophilicity was analyzed by using static water contact angle measurements (DataPhysics, Germany), applying the Young–Laplace fitting procedure. Scanning electron microscopy (SEM, Zeiss, Germany) was used to assess surface topography. SEM images were acquired at an average working distance of 9 mm and an accelerating voltage of 5 kV. Atomic force microscopy (AFM) measurements were carried out on an alpha300 RA microscope from WITec (Oxford Instruments) in tapping mode. An AFM “Arrow Cantilever” (resonance frequency of the tip: approximately 275 kHz, spring constant 42 N/m) from WITec was used. Roughness parameters were calculated with Gwyddion Version 2.58.

### Cell Culture and Cell Vitality Assay

Two cell types were used in this study: NIH/3T3 fibroblasts (CLS GmbH, Eppelheim, Germany) and HUVEC (PromoCell, Germany). Mouse embryonic fibroblasts were cultured as a monolayer in T75 tissue culture flasks (Greiner Bio-One GmbH) using Dulbecco’s Modified Eagle’s Medium (DMEM) - high glucose (4.5 g/L glucose) (Life Technologies GmbH), supplemented with 10% fetal calf serum (FCS) (Thermo Fisher Scientific, Germany) and 1% penicillin/streptomycin (Pen/Strep) (Life Technologies GmbH). HUVECs were cultured in T75 tissue culture flasks (Greiner Bio-One GmbH) using endothelial cell growth medium (PromoCell, Germany) at 37 °C in 5% CO_2_, with an initial seeding density of 5000 cells/cm^2^.

For the cell adhesion and proliferation tests, the PEM- and PEM/Wax-coated wells of 24-well cell culture plates were seeded with 50,000 cells/mL and incubated at 37 °C in 5% CO_2_ for 24, 48, and 72 h, respectively. As a positive control, commercial tissue culture polystyrene plates (TCPS, Greiner Bio-One GmbH) were used, and as a negative control, uncoated polystyrene well plates (Greiner Bio-One, Germany) were employed. To assess cell viability and proliferation, a resazurin assay was performed. After 24, 48, and 72 h of cultivation, phase contrast images of the adhered cells were first acquired at 10× magnification to evaluate cell morphology and confluency. The cell culture medium was then replaced with fresh medium containing 10% resazurin (Sigma-Aldrich GmbH) and incubated for 6 h at 37 °C. During this time, viable cells metabolized and reduced resazurin to resorufin, resulting in a color change from blue to fluorescent violet. Absorbance was measured at 574 (resazurin) and 604 nm (resorufin) using a microtiter plate reader (Safire II-Basic; from Tecan Austria GmbH).

### In Vitro Cytotoxicity Assay

The biocompatibility of the basic PEM and composite PEM/Wax coatings was tested in triplicate under sterile conditions, following ISO 10993-5, by extracting potentially cytotoxic substances that may be released under in vivo implantation conditions. Extractions were carried out at 37 °C for 24 h, as per the standard protocol, with continuous agitation. The extracting medium used was DMEM supplemented with 10% FCS and 1% PEN/Strep (ISO 10993-12). Cytotoxic latex and noncytotoxic polypropylene (PP) were used as positive and negative controls, respectively. The extracts were used immediately for subsequent biological tests.

NIH/3T3 cells were seeded at a concentration of 10,000 cells/well in 96-well microplates and incubated for 24 h at 37 °C in a 5% CO_2_ atmosphere. After incubation, the medium was replaced with the extracts. As recommended in ISO 10993-5, a series of dilutions of the extraction medium with fresh culture medium was prepared and applied to the subconfluent cell layer. Following a 24 h incubation period, cell vitality was assessed using the resazurin reduction assay.

### Statistical Analysis

All statistical analyses were performed using Microsoft Excel. Data are presented as the mean ± standard deviation (SD) unless otherwise stated. To assess differences between different data groups, a one-way analysis of variance (ANOVA) was conducted. A *p*-value of less than 0.05 was considered statistically significant.

## Results and Discussion

### Physicochemical Characteristics of PEM and PEM/Wax Coatings

The successful adsorption of the hydrophobic wax nanoparticles onto the hydrophilic PEM matrix, and vice versa, during the stepwise construction of the composite PEM/Wax coatings was demonstrated by monitoring the thickness and static water contact angle values during the construction of the coatings, after each material block was deposited.

Regardless of the type of PEM used as a base for wax adhesion, the coating thickness increased after each deposited building block, confirming the success of each addition of a material (Figure 2A). The thickness of the base PEM coatings ranged from 2.6 to 20.4 nm, depending on their chemical nature, while their composite counterparts with two wax layers measured between 94 and 105 nm. Thus, all coatings are nano-thick and practically do not reshape the substrate. The thickness of PSS/PAH/Wax films increases linearly, similar to the growth behavior of PSS/PAH films.^23^ In contrast, the HA/Chi/Wax coating exhibited exponential growth, mirroring the characteristic growth pattern of HA/Chi multilayers.^24^ The very low thickness of the (PAA/PAH)_7_ multilayer is consistent with previous studies, which showed that the properties and growth mechanism of this PEM are highly sensitive to the pH of the polyelectrolyte solutions used during construction. At pH 7.0, as applied in this study, the thickness of the adsorbed PAA and PAH monolayers is only 0.1 nm.^25,26^

**Figure 2:**
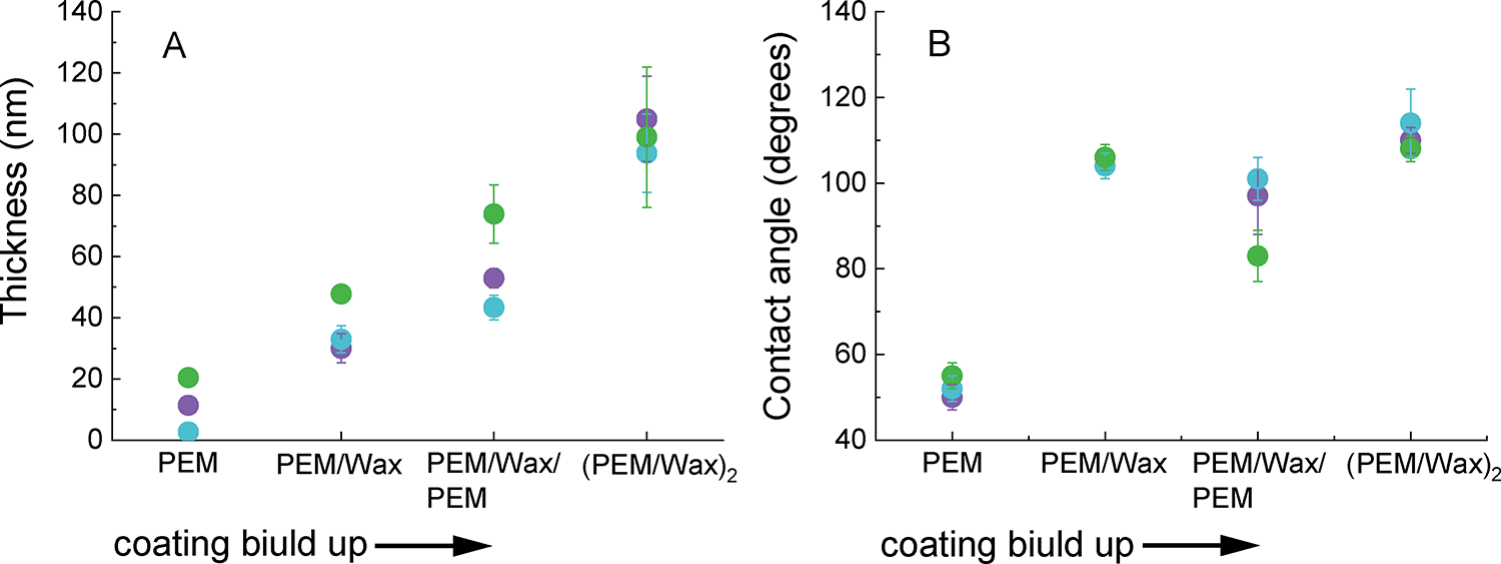
Evolution of thickness (A) and water contact angle (B) during the stepwise construction of the three composite PEM/Wax coatings. PEM refers to hyaluronic acid/chitosan (HA/Chi, purple circles), poly­(acrylic acid)/poly­(allylamine hydrochloride) (PAA/PAH, blue circles), and polystyrenesulfonate/poly­(allylamine hydrochloride) (PSS/PAH, green circles).

As the films become thicker, the standard deviation of the thickness also increases (Figure 2A). This may be attributed to the increased surface roughness of the coatings following the deposition of the wax layers, as suggested by the SEM and AFM images of the coating morphologies shown in Figures 3 and 4 and listed in Table 1.

**Figure 3:**
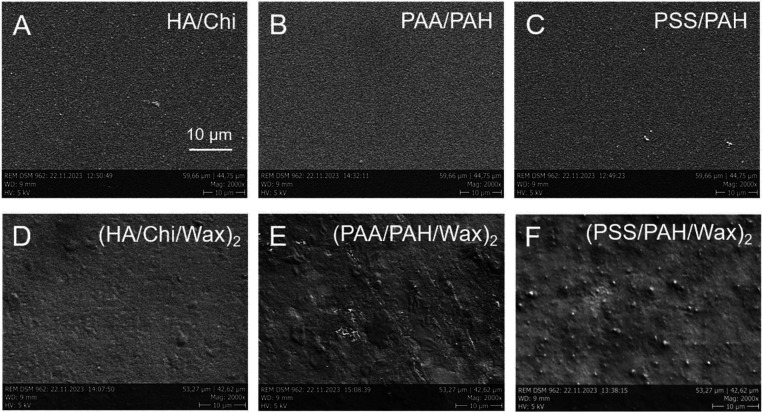
SEM surface morphology images of base PEMs (A–C) and their corresponding composite (PEM/Wax)_2_ films (D–F).

**Figure 4:**
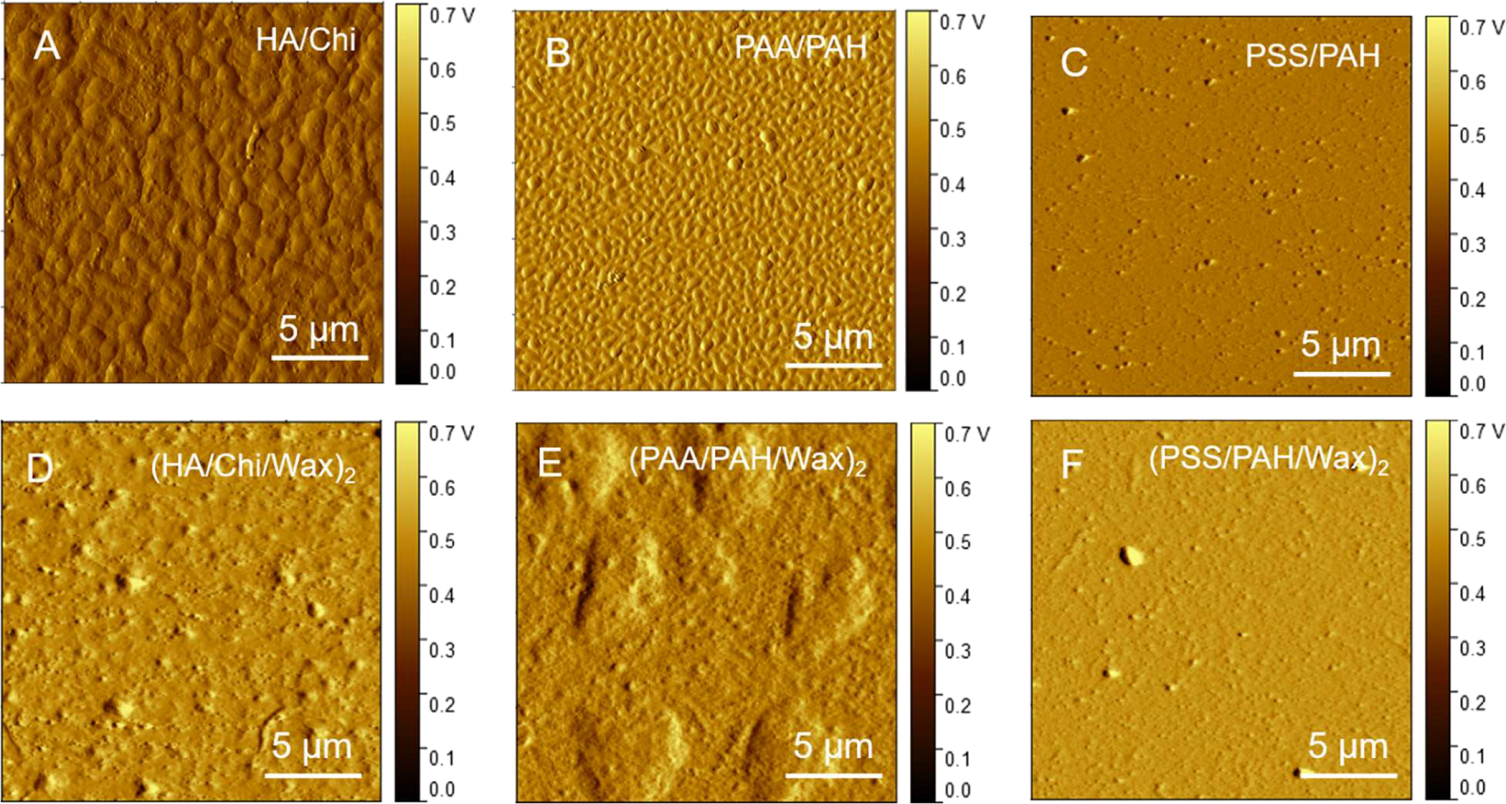
AFM amplitude images of base PEMs (A–C) and their corresponding composite (PEM/Wax)_2_ films (D–F).

**Table 1:** Arithmetic Mean Surface Roughness (Sa) and Root Mean Square Roughness (Sq) of the PEM and PEM/Wax Coatings as Evaluated by AFM^a^

(HA/Chi)_7_	(PAA/PAH)_7_	(PSS/PAH)_7_
Sa 6.6 ± 0.6 nm	Sa 4.7 ± 0.5 nm	Sa 2.2 ± 0.1 nm
Sq 8.2 ± 0.9 nm	Sq 6.4 ± 0.8 nm	Sq 4.0 ± 0.4 nm

(HA/Chi/Wax)_2_	(PAA/PAH/Wax)_2_	(PSS/PAH/Wax)_2_
Sa 14.8 ± 0.3 nm	Sa 34.2 ± 7.1 nm	Sa 8.0 ± 2.1 nm
Sq 21.3 ± 1.2 nm	Sq 45.0 ± 9.1 nm	Sq 16.6 ± 5.5 nm

a Data are presented as the mean value ± standard deviation.

Both the SEM images (Figure 3) and the AFM images (Figure 4) demonstrate that the changes in surface morphology induced by the hydrophobic wax layers are PEM-specific. Among the three multilayers, PSS/PAH films display the smoothest surfaces (Figure 4C), and this trend is preserved in their wax-modified counterpart (PSS/PAH/Wax) (Figure 4F). In contrast, the HA/Chi and PAA/PAH multilayers both exhibit irregular surface protrusions (Figure 4A,B). Notably, these features disappear in the HA/Chi/Wax films (Figure 4D), whereas in PAA/PAH/Wax films, they persist and increase markedly in size (Figure 4E).

All three base PEMs are hydrophilic, with average contact angles ranging from 50 to 55°. In contrast, all corresponding composite PEM/Wax and (PEM/Wax)_2_ coatings are hydrophobic, exhibiting contact angles between 105 and 115° (Figure 2B). This confirms that regardless of the type and properties of the base PEM, wax particles self-adhere effectively from the suspension, forming a homogeneous wax layer, as indicated by the relatively low standard deviation in the contact angle measurements. Furthermore, the water contact angles of the first and second wax layers are nearly identical and appear to be independent of the type of underlying PEM. An interesting observation is that the PEM/Wax/PEM coatings, when terminated with PEM films, are more hydrophobic than the PEM films alone. This can be due to the fact that the initial PEM blocks were deposited on a hydrophilic substrate (Si-wafer or TCPS polystyrene well plate), whereas the second PEM blocks were built upon a hydrophobic wax layer. This observation aligns with previous findings, which reported that PAA/PAH multilayers with fewer than eight bilayers, built on a hydrophobic self-assembled octadecyltrichlorosilane (ODTS) layer (with a contact angle ∼108°), exhibited significantly higher hydrophobicity (water contact angle up to 95°) than those constructed on a silicon substrate.^27^ Another possible explanation for this observation lies in the Cassie model, which relates the contact angle to the surface roughness. According to this model, as the roughness of a given surface increases, the contact angle likewise increases.^28^ The surface roughness data of the coatings, presented in Table 1, indicate that the introduction of wax layers does increase the roughness. However, an increase of only a few nanometers is insufficient to nearly double the contact angle. Therefore, in this case, surface chemistry plays a far more significant role than such a minimal roughness change.

### In Vitro Cell Adhesion and Proliferation on the PEM and Composite PEM/Wax Coatings

In this study, both fibroblasts and HUVECs were employed to evaluate cell behavior on composite hydrophobic coatings. Fibroblasts, originating from connective tissue, are primarily involved in extracellular matrix production and tissue remodeling, whereas HUVECs represent vascular endothelial cells that are responsible for lining blood vessels and regulating angiogenesis and hemostasis. Their inclusion enables a broader assessment of the biocompatibility of hydrophobic surfaces in contexts where implants may interact with both connective and vascular tissues, for example, in vascular stents. Moreover, fibroblasts and HUVECs often exhibit differing responses to surface hydrophobicity, chemistry, and topography, making them suitable for identifying cell-type-specific interactions, which is the goal of this study. Such comparative analysis not only provides insights into the selective promotion or inhibition of adhesion and proliferation but also supports the optimization of coating properties for targeted biomedical applications.

In actively proliferating, healthy cell cultures grown under standard, nonstressed conditions, the resazurin assay provides a reliable measure of metabolic activity, which typically correlates well with viable cell number. In such cases, higher confluence observed in microscopy images generally reflects a greater number of metabolically active cells.^29^ However, while the resazurin assay yields a quantitative average of the metabolic activity across the entire well, microscopy offers localized, qualitative insights, capturing details such as cell morphology, spreading, and surface distribution. It is important to note that nonuniform cell distribution can result in regions of high confluence alongside sparsely populated areas. This spatial variability may complicate the correlation between the visual confluence and overall viability measured by resazurin. Moreover, resazurin reduction is influenced not only by cell number but also by the metabolic state of the cells. Cells experiencing stress or progressing through different phases of the cell cycle may exhibit altered metabolic activity, which can affect resazurin reduction independently of cell density.^30,31^ Notably, under certain stress conditions, cells may upregulate NADH production as part of survival or adaptive responses, potentially leading to an overestimation of viability in metabolic assays.^30,31^

Therefore, to obtain a comprehensive and accurate interpretation of cell adhesion and growth behavior in this study, we employed both resazurin-based viability assays and microscopy imaging in a complementary manner. While the resazurin assay quantified overall metabolic activity, imaging allowed us to assess cell morphology, spreading, and localized density patterns, offering a fuller picture of the cell-material interactions.

The data in Figure 5 and the conducted statistical analysis showed that the ability of the three base PEM and three composite PEM/Wax coatings to support cell adhesion and viability after 24 h of incubation follows this order: HA/Chi ≈ PAA/PAH ≈ PSS/PAH < HA/Chi/Wax ≈ PAA/PAH/Wax ≈ PSS/PAH/Wax ≈ PC. Therefore, fibroblasts show better viability on the hydrophobic composite PEM/Wax coatings than on the hydrophilic PEM coatings of the same type.

**Figure 5:**
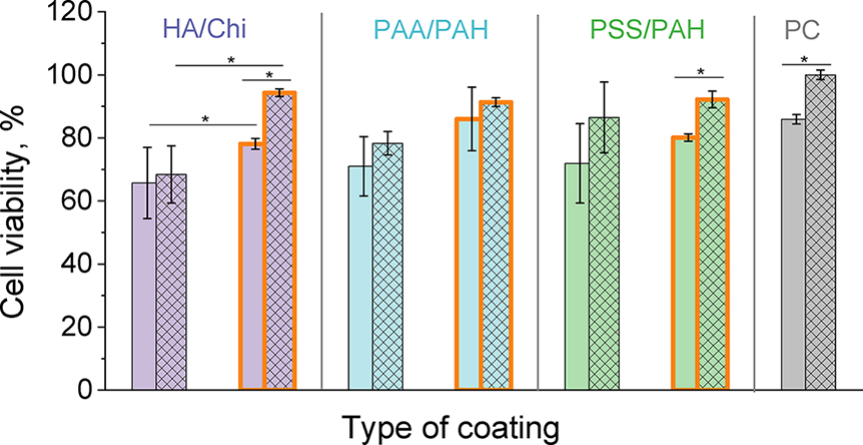
Viability of 3T3 fibroblasts on the three base PEM coatings hyaluronic acid/chitosan (HA/Chi, purple columns), poly­(acrylic acid) /poly­(allylamine hydrochloride) (PAA/PAH, blue columns), and polystyrenesulfonate/poly­(allylamine hydrochloride) (PSS/PAH, green columns), and their corresponding composite (PEM/Wax)_2_ coatings (orange framed columns) after 24 (solid columns) and 72 h (hatched columns) of cultivation, relative to the positive control (PC, TCPS plate). (*p < 0.05) Measurements at 48 h are consistent with the trends observed at 24 and 72 h. As these intermediate results did not provide additional insights beyond the presented time points, they are not shown here to maintain clarity and focus in the figure.

Numerous studies have collectively demonstrated the anticell-adhesive properties of hydrogel-like HA/Chi coating. Reduced cell attachment and proliferation on HA/Chi coatings have been observed in a variety of cell types, including A549 epithelial cells (human lung carcinoma line),^32^ BHK fibroblasts (from baby hamster kidney),^32^ C2C12 mouse myoblasts,^32^ MC-3T3-E1 murine osteoblasts,^32^ human platelets,^24,33^ HUVECs,^34,35^ mouse embryonic fibroblasts 3T3,^34^ and HCS-2/8 human chondrosarcoma cells.^36^ The present study aligns with these findings, as evidenced by reduced cell viability (Figure 5) and the morphological appearance of the cells in Figure 6, where fibroblasts on HA/Chi-functionalized surfaces appear round and clustered, in contrast to the normal well-spread morphology observed on PAA/PAH- and PSS/PAH-functionalized surfaces.

**Figure 6:**
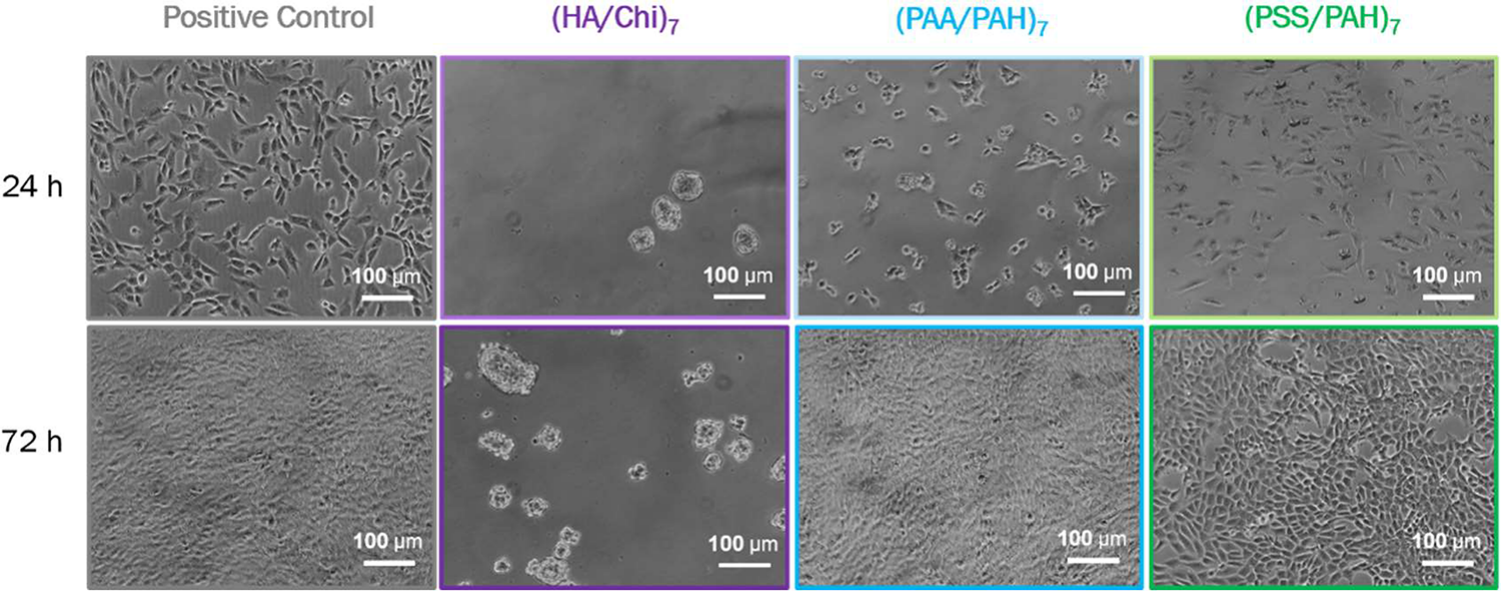
Microscopic images of 3T3 fibroblasts on the three base PEM coatings and the positive control after 24 and 72 h of incubation.

The incorporation of wax layers and surface hydrophobization has a significant cell-promoting effect on fibroblasts, particularly in the case of HA/Chi coating (Figure 5). 3T3 cells on the HA/Chi/Wax coating exhibit a well-spread morphology (Figure 7), indicating good adhesion to the surface, which leads to substantial proliferation over time, reaching 95% relative to that of the TCPS control.

**Figure 7:**
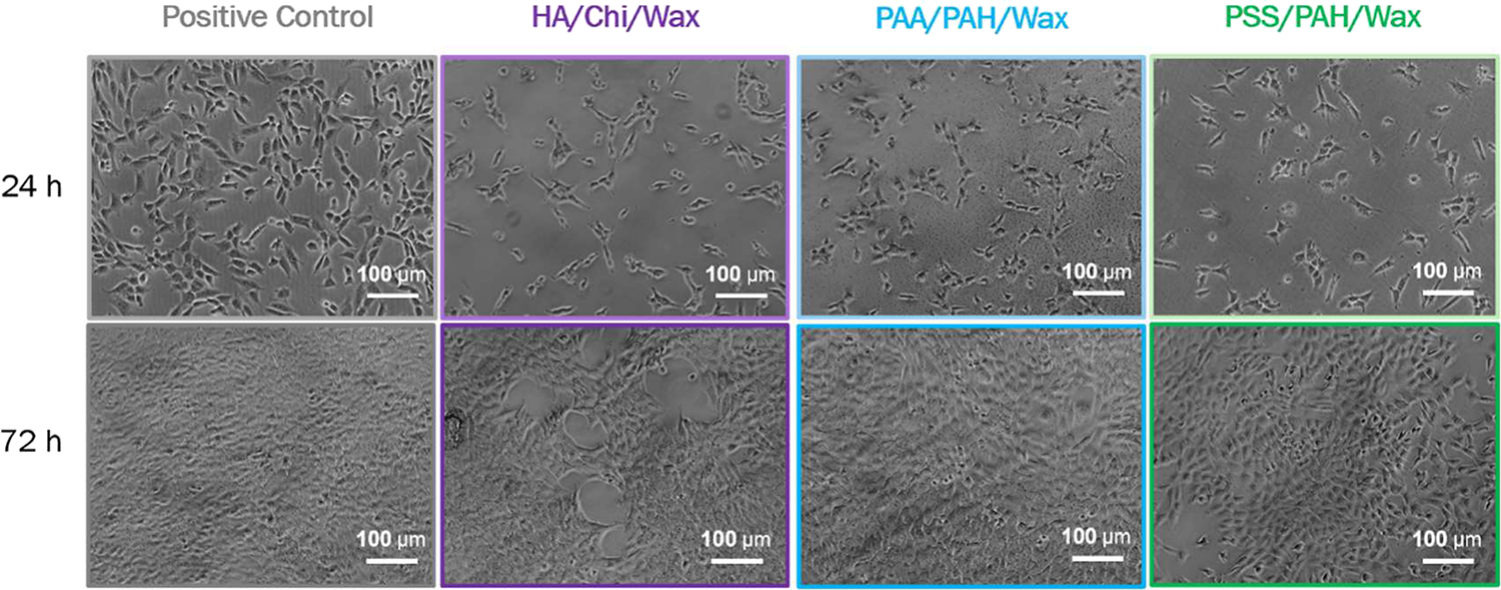
Microscopic images of 3T3 fibroblasts on the three composite (PEM/Wax)_2_ coatings and the positive control after 24 and 72 h of incubation.

An important finding is that all composite (PEM/Wax)_2_ coatings promote fibroblast viability that is statistically equivalent (*p* < 0.05), reaching up to 95% relative to the gold standard, TCPS. This supports the notion that cellular behavior on a specific surface is largely governed by surface properties,^37^ rather than the bulk characteristics of the material. Cells “sense” and respond primarily to the outermost layer of the substrate with which they are in direct contact. In this case, the thin external wax layer dictates fibroblast viability, overshadowing the influence of the underlying polymer matrix’s chemical composition.

Although fibroblasts typically exhibit decreased adhesion on hydrophobic surfaces compared to hydrophilic surfaces, this trend is not absolute. Hydrophobicity can limit initial cell attachment and spreading by reducing protein adsorption and weakening cell-substrate interactions.^38^ However, fibroblasts may still adhere to moderately hydrophobic surfaces, albeit less efficiently than on hydrophilic substrates.^39^ The equilibrium between surface chemistry and the biological environment strongly influences fibroblasts' behavior, and surface modifications, such as protein precoating, can sometimes enhance cell adhesion even on hydrophobic surfaces.^39^

The Berg limit, proposed by Vogler et al.^40^ is a widely accepted threshold for understanding the influence of surface hydrophobicity or hydrophilicity on protein adsorption phenomena. According to Vogler’s classification, surfaces with a contact angle greater than 65° are considered hydrophobic, while those with a contact angle below 65° are classified as hydrophilic. Vogler’s theory, which has been validated both experimentally and theoretically over the years,^41^ suggests that hydrophobic surfaces possess a greater capacity for protein adsorption than hydrophilic ones. It was hypothesized that water molecules are tightly bound to hydrophilic surfaces, making it difficult for proteins to displace them via surface dehydration mechanisms. Applying this theory to the data on coating hydrophilicity shown in Figure 2B, it is evident that the contact angles of the base PEM films fall slightly below the 65° threshold, while they increase dramatically upon hybridization with wax layers. The observed increase in fibroblast adhesion and proliferation on PEM/Wax coatings, as compared to the base PEMs, is presumably due to altered protein adsorption from serum-containing culture medium (10% FCS), driven by changes in surface hydrophobicity.

In addition to enhancing surface hydrophobicity, hybridization with wax layers significantly increases the surface roughness of the otherwise nanosmooth PEM films^17,42^ and introduces distinct surface patterning and structuring (Figure 3). Moderate surface roughness is known to promote fibroblast adhesion and proliferation by increasing the available surface area for cell attachment, providing mechanical interlocking sites, and offering topographical cues for focal adhesions. Moreover, such roughness better mimics the natural extracellular matrix, further facilitating cell attachment.^43,44^ For example, surfaces with microgrooved titanium have shown enhanced fibroblast adhesion and activation compared to polished surfaces.^43^ Another study reported that while polished surfaces (average roughness ∼ 60 nm) exhibited stronger initial fibroblast attachment, rougher surfaces (average roughness ∼ 220 nm) supported significantly greater proliferation.^44^ Additionally, polishing titanium and titanium alloy implants has been shown to reduce cell and tissue adhesion in vivo.^45,46^ A similar trend was observed in our study: all nanosmooth PEM coatings showed no statistically significant increase in cell viability between 24 and 72 h. In contrast, the rougher HA/Chi/Wax and PSS/PAH/Wax coatings demonstrated a statistically significant increase in cell proliferation over the same period (Figure 5).

The behavior of HUVECs on hydrophilic PEM and hydrophobic PEM/Wax coatings of various chemical compositions differs significantly from that of the 3T3 fibroblast. HUVECs line blood arteries and play a key role in regulating blood flow, vascular permeability, and response to vascular injury. Compared to fibroblasts, which are more resilient to physical changes and spatial constraints, HUVECs are more sensitive to alterations in their environment, including the presence of growth factors, toxins, or mechanical stress.^47^ Several studies have shown that HUVECs exhibit a stronger response than fibroblasts to certain external stressors, such as chemical exposures. For instance, they are more susceptible to compounds like perfluorooctanesulfonic acid (PFOS), which impacts their metabolism more significantly than that of fibroblasts.^47^

The viability of HUVECs shows a stronger dependence on the chemical composition of the base PEM coatings compared with that of fibroblasts (Figure 8). While their viability increases in the same order as for fibroblasts, the impact of the different PEMs is more pronounced. PSS/PAH is the most cell-compatible coating, supporting HUVECs' viability of 98% relative to TCPS, in contrast to HA/Chi coating, which provides less favorable conditions for cell attachment and growth.

**Figure 8:**
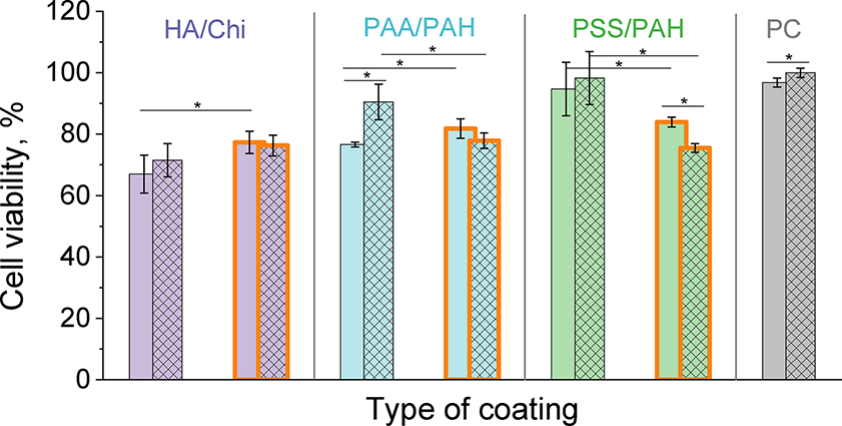
HUVECs viability on the three base PEM coatings hyaluronic acid/chitosan (HA/Chi, purple columns), poly­(acrylic acid) /poly­(allylamine hydrochloride) (PAA/PAH, blue columns), and polystyrenesulfonate/poly­(allylamine hydrochloride) (PSS/PAH, green columns), and their corresponding composite (PEM/Wax)_2_ coatings (orange framed columns) after 24 h (solid columns) and 72 h (hatched columns) of cultivation, relative to the positive control (PC, TCPS plate). (*p < 0.05) Measurements at 48 h are consistent with the trends observed at 24 and 72 h. As these intermediate results did not provide additional insights beyond the presented time points, they are not shown here to maintain clarity and focus in the figure.

The initial adhesive interaction between cells and the substrate is influenced by the surface’s chemical composition and charge. Both PSS/PAH and PAA/PAH, which support over 90% cell viability, are terminated with PAH, exposing primary amino groups at the surface. Our findings are consistent with earlier research showing that amine-functionalized coatings can enhance cell attachment, proliferation, and differentiation.^48^ For example, prior studies treated PTFE and polyethylene terephthalate (PET) with a mixture of ammonia and ethylene in a radiofrequency plasma reactor.^49^ The results demonstrated improved HUVEC adhesion and growth on plasma-treated surfaces compared to those on untreated ones. In another study, unmodified PTFE and PTFE modified with a layer of plasma-polymerized allylamine were used to investigate early HUVEC adhesion.^50^ Statistical analysis revealed significantly enhanced HUVEC adherence on the plasma-polymerized allylamine films compared to the bare PTFE.

In the case of HUVECs, surface hydrophobization slightly improves cell adhesion on HA/Chi-functionalized surfaces and has no significant effect on PAA/PAH surfaces but markedly suppresses adhesion on PSS/PAH-coated surfaces, thereby equalizing HUVEC viability across all composite coatings (Figure 8). Prolonged incubation on hydrophobic surfaces for 72 h further reduces cell viability. These results are supported by the microscopic images in Figures 9 and 10, which show spindle-shaped endothelial cells on hydrophilic PSS/PAH and PAA/PAH coatings, rounded and clustered cells on hydrogel-like HA/Chi coatings, and a time-dependent transition from spread to rounded morphology on the hydrophobic PEM/Wax surfaces.

**Figure 9:**
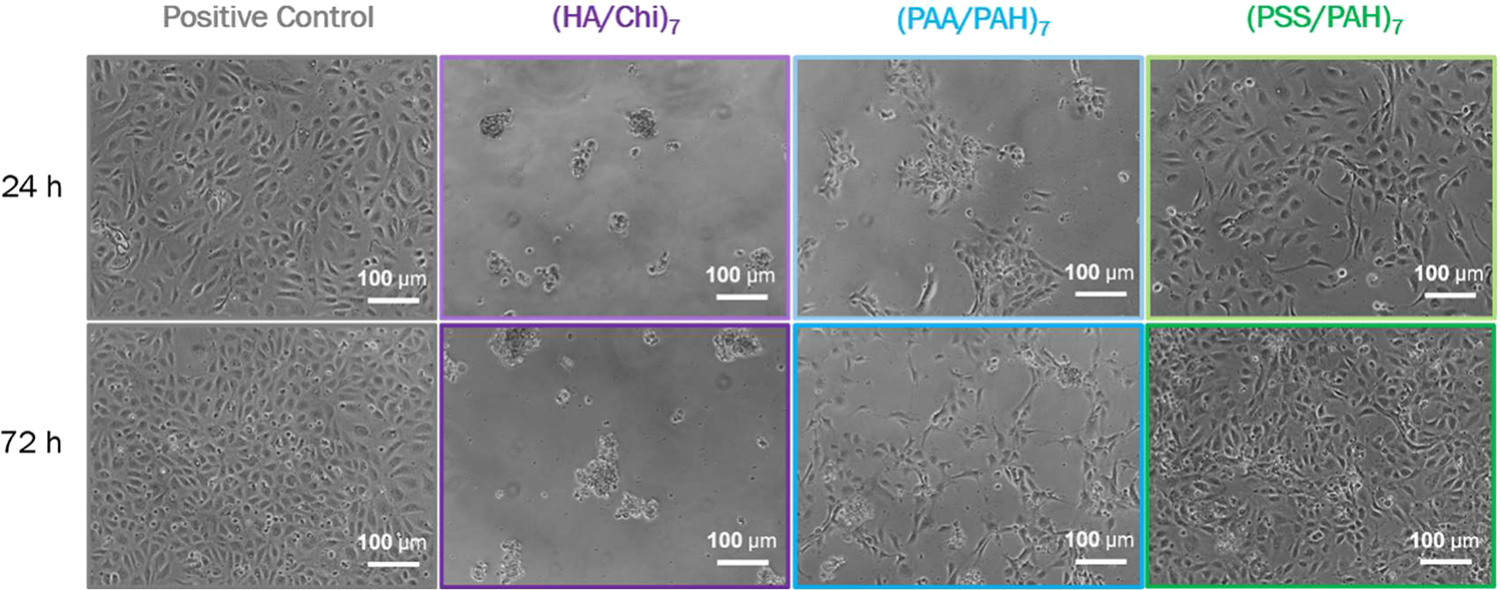
Microscopic images of HUVECs on the three base PEM coatings and the positive control after 24 and 72 h of incubation.

**Figure 10:**
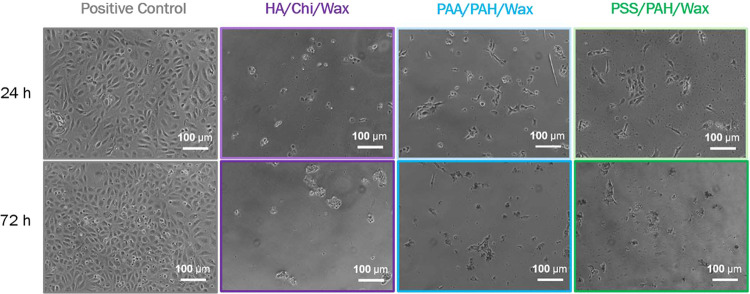
Microscopic images of HUVECs on the three composite (PEM/Wax)_2_ coatings and the positive control after 24 and 72 h of incubation.

It has been observed that cellular responses to surface roughness are cell-type specific. For instance, endothelial, epithelial, and periodontal fibroblast cells generally adhere and spread more effectively on smooth surfaces, whereas osteoblasts show a preference for rougher textures.^51^ Endothelial cells are more sensitive to changes in roughness than fibroblasts. It is widely accepted that low-scale roughness in the range of 10–200 nm is typically beneficial for their adhesion and function, while excessive or irregular surface roughness can hinder adhesion, reduce viability, and compromise cellular function.^52^ In this study, the results for HUVECs were not unequivocal, indicating that surface roughness alone cannot account for differences in cell viability. Other factors, such as surface hydrophilicity and chemical composition, also play critical roles. An increase in roughness on the HA/Chi/Wax coatings resulted in a statistically significant increase in HUVEC viability after 24 h. In contrast, a similar increase in roughness on the PSS/PAH/Wax coatings led to a decrease in cell viability, underscoring the importance of multiple surface parameters in influencing endothelial cell behavior.

### Cytotoxicity of PEM and PEM/Wax Coatings

Since the coatings in this study are intended for use in medical devices, cytotoxicity testing was performed to demonstrate their safety in accordance with the requirements of the regulatory standard ISO 10993-5, which certifies the safety of medical devices for clinical use.

The growth inhibition test, conducted using 3T3 mouse fibroblasts, revealed that extracts from both the PEM and PEM/Wax coatings exhibited no marked cytotoxic activity (Figure 11). No release of toxic substances that could inhibit cell growth was detected. The highest observed cell mortality was 12% (for the PSS/PAH multilayer at 100% extraction medium), which is considered noncytotoxic according to the guidelines of ISO 10993-5.

**Figure 11:**
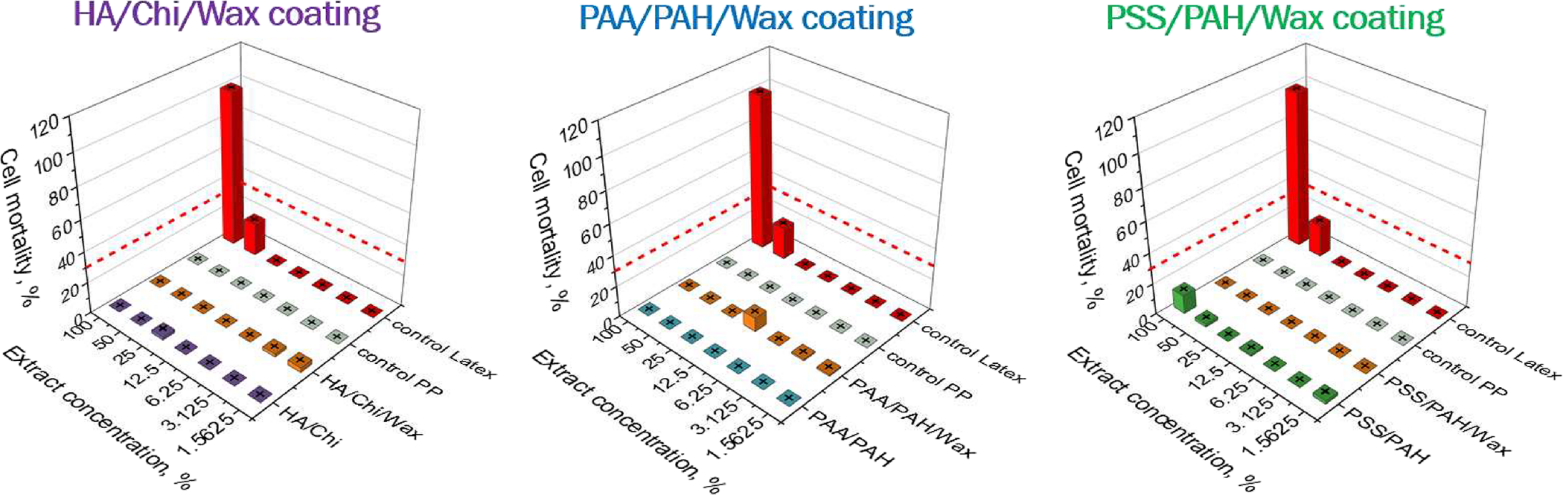
Cell mortality of 3T3 fibroblasts as a function of the concentration of cell culture medium extracts from base PEM and composite PEM/Wax coatings. Latex was used as a positive control, and polypropylene as a negative control.

Figure 11 shows the relative mortality of 3T3 cells exposed to a series of dilutions of extracts from the three PEM and three PEM/Wax coatings, under investigation, alongside the positive (cytotoxic latex) and negative (noncytotoxic PP) controls. As expected, the cytotoxic latex caused 100% inhibition of cell growth at high concentrations with decreasing toxicity upon dilution. In contrast, the noncytotoxic PP showed 0% mortality across all concentrations. According to ISO 10993-5, a material is classified as noncytotoxic if the extract at maximum concentration results in a reduction of cell viability by 30% or less. Based on this criterion, all coatings tested in this study are considered noncytotoxic, as they do not exhibit any significant growth-inhibiting effect on 3T3 mouse fibroblasts.

## Conclusions

This study was motivated by prior findings demonstrating that composite PSS/PAH/Wax coating, consisting of alternating PSS/PAH matrices and nanothin wax layers, exhibits strong hydrophobicity. When applied to the surface of commercial Mg-based coronary stents, this coating significantly delayed in vitro biodegradation. Building on this concept, in this work, we extended the approach to construct two additional coatings: PAA/PAH/Wax and HA/Chi/Wax. Our results show that the method for constructing hydrophobic PEM/Wax composite coatings is both robust and versatile. Despite their hydrophobic nature, all three PEM/Wax coatings supported the adhesion and growth of 3T3 fibroblasts, outperforming their respective hydrophilic PEM counterparts. In contrast, HUVECs, which are more sensitive to surface and environmental cues, displayed reduced adhesion and viability on the hydrophobic PEM/Wax surfaces compared to those of their hydrophilic PEMs. While the cellular behavior on the PEM coatings alone was highly dependent on the specific PEM composition and its surface properties, the composite PEM/Wax coatings induced a more uniform cellular response across all types tested. The cytotoxicity evaluation of the coatings, carried out in accordance with ISO 10993-5, showed that they are noncytotoxic. Given their hydrophobicity, biocompatibility, and barrier properties, PEM/Wax coatings hold promise for applications requiring antifouling, anticontamination, and moisture-barrier properties, such as wound dressings, biomedical sensors, and protective coatings for implantable bioresorbable materials.
